# Technology Guided Management of Orbital Trauma: A Systematic Review

**DOI:** 10.1007/s12663-024-02255-9

**Published:** 2024-08-01

**Authors:** Luis Vicente González, Juan Pablo López, María Paula Orjuela, David Díaz-Báez

**Affiliations:** 1 Oral and Maxillofacial Surgeon, Fundacion Santa Fe de Bogotaá, Bogotá, Colombia; 2 Department of Oral Research. School of Dentistry, Institución Universitaria de Colegios de Colombia UNICOC, Bogotá, Colombia; 3 Oral and Maxillofacial Surgeon, Hospital Universitario Fundación Santa Fe de Bogotá, Calle 129#7d-47, Bogotá, Colombia; 4 Universidad El Bosque, Unidad de Investigación en Epidemiología Clínica Oral UNIECLO, Calle 129#7d-47, Bogotá, Colombia; 5 Oral and Maxillofacial Surgeon, Fundacion Santa Fe de Bogotaá, Bogotá, Colombia; 6 Unit of Basic Oral Investigation (UIBO), Facultad de odontología, Universidad El Bosque, Bogotá, Colombia

**Keywords:** Endoscopic support, Virtual surgical navigation, Patient-specific implant, Virtual surgical planning

## Abstract

**Background:**

The use of technology in the surgical field has been increasing; however, the literature that studies the combination of these techniques is still scarce.

**Purpose:**

This systematic review aims to identify the combination of the different technological tools currently available for surgical reconstruction of the orbit after trauma injury and determine the most common treatment alternatives.

**Methods:**

A search following PRISMA criterios was carried out of articles published between January 2015 and December 2019 in PubMed, Embase, Scopus, and Ovid databases. The Mesh terms were orbit fracture, orbit trauma, computer-assisted surgery, surgical navigation system, navigation surgery, endoscopic surgical procedure, endoscopy, endoscope support, and patient-specific implants. The inclusion criteria were orbital trauma, articles that described the combination of technological tools, cases, case series, retrospective studies, and randomized clinical trials. Pediatric trauma management studies were excluded. To determine methodological quality and risk of bias the Joanna Briggs Institute Verification List (JBI) was used. The results were collected and presented in tables for easy interpretation.

**Results:**

Of the articles found, 12 were finally chosen. Most of the articles (8) were case series, 2 were case reports, 1 was quasi-experimental, and 1 was a randomized clinical trial. A total of 418 patients were reported in all studies, and the most widely used tool was virtual planning, reported in 11 articles (91.6%). Virtual surgical planning in combination with intraoperative navigation was adopted by 9 articles (75%), being the most used combination of technologies.

**Conclusions:**

Integration of virtual surgical planning, intraoperative navigation, patient-specific implants, and endoscopic techniques will help to improve the results significantly in the initial management of the orbital trauma. However, it is recommended in future studies that the results be evaluated in the same way to obtain more homogeneous studies.

## Introduction

When managing orbital fractures, there are still controversies that include adequate time for reconstruction, the surgical approach, and the reconstruction materials that are available for use in these procedures. Nowadays, the increasing use of technology adds to the confusion [1].

Virtual surgical planning (VSP) in the orbit has great relevance due to the complex anatomy. This is why it can increase the precision of the placement of the implant material and the preservation of neurovascular structures in the area [2].

In 1972, Walter published the use of endoscopic support through a trans-anthral approach for the early repair of orbital fractures [3]. Orbital reconstruction must respect the neurovascular structures, and endoscopy has been shown to be helpful when used in minimally invasive approaches, allowing for direct visualization of critical structures and being less disruptive of the anatomy when compared to conventional techniques.

Virtual intraoperative navigation in reconstruction has inconveniences such as cost and additional training [4]. But currently, it allows planning the procedure, delimiting the defect, and applying volumetric tools that improve precision [5]. This provides an intraoperative guide in real-time, which is more precise and accurate, allowing proper positioning of the implants [6].

Patient-specific implants (PSI) are being widely used, and the advantages of their use are mainly based on precision and correct attachment to bone defects, which reduces the rate of surgical reoperation [7, 8]. Besides, it has also been demonstrated that intraoperative times are reduced, which in turn can reduce complications [9]. It is recommended to complement the surgical technique for large fractures or when multiple orbital walls are involved [10].

Although the use of technology in the surgical field has been increasing, the literature that studies the combination of these techniques is still scarce. Therefore, this systematic review aims to evaluate which is the most common combination of technologies for the treatment of orbital trauma by measuring variables that evaluate both aesthetic and functional results.

## Methods

This protocol has been registered in the PROSPERO database with ID CRD42021250376, following the PRISMA criteria under the following research question:

P: Adult patients over 18 years with orbital fractures that require surgical reduction.
I: Orbital fracture reduction.
C: Orbital fracture reduction using different combination of technological tools
  (each of the combinations mentioned in the reviewed articles).
O: Impact on the improvement evaluated by both functional and aesthetic variables.

### Criteria for Selecting Studies

#### Inclusion Criteria

Articles in English published between January 2015 and December 2019 that only reported orbital trauma described the combination of technological tools used (clinical cases, case series, observational studies, and randomized clinical trials).

#### Exclusion Criteria

Pediatric trauma and resections for malignant lesions were excluded.

#### Types of Technological Tools

- Endoscopic Support
- Virtual Planning
- Virtual Navigation
- Patient-specific implant or Rapid Prototyping

### Search Methods for Identification of Studies

A literature search was performed in Medline (via PubMed), EMBASE (via OVID), and Scopus. The key terms in natural language and controlled language were identified for the conditions and interventions of interest. A generic search strategy composed of controlled vocabulary, Mesh (Medical Subject Headings), and free language, considering synonyms, abbreviations, acronyms, spelling, and plural variations, was designed. Individual search strategies were developed for each source of information. This step was complemented by a search for additional publications using the snowball methodology (Appendix 1).

#### Critical Appraisal and Assessment of Risk of Bias

All studies except for one were evaluated independently and duplicated by two reviewers (JPL & MPO) to determine methodological quality using the Joanna Briggs Institute Verification List (JBI) for cross sectional designs/Case Cases and Controls,/Case Report, Appendix 2). The JBI checklists for the case series corresponded to ten questions requiring a yes, a no, or an unclear answer. All the studies that obtained more than seven "yes" were considered to have adequate methodological quality; those that presented at least five were considered acceptable; and those with fewer than five were considered low quality. The JBI checklists for case reports corresponded to eight questions requiring a yes, a no, or an unclear answer. All the studies that obtained more than six "yes" were considered to have adequate methodological quality, those that presented at least four were considered acceptable; and those with fewer than four were considered low quality. The JBI checklists for quasi-experimental corresponded to nine questions requiring a yes, a no, or an unclear answer. All the studies that obtained more than seven "yes" were considered as adequate methodological quality; those that presented at least five were considered acceptable; and those with fewer than five were considered low quality. One study was evaluated using the ROB 2 tool (Version 2 of the Cochrane risk-of-bias tool) for randomized clinical trials. This tool is based on the evaluation and qualification of clinical studies, considering 5 domains where it focuses on assessing different aspects (trial design, conduct, and reporting) that are relevant to the risk of bias in a study of this type. Disagreements related to the rating of the studies among the reviewers, were subjected to the evaluation of a third evaluator (LG).

### Data Collection

The list with the bibliographic references identified in the electronic searches was downloaded into a library of the Rayyan® program, where duplicate publications were eliminated, and an initial screening was carried out. Three of the authors initially assessed the abstracts separately and selected potentially eligible studies. The three reviewers subsequently independently verified the eligibility criteria (inclusion and exclusion) by reviewing each full-text publication. All disagreements were solved by consensus.

### Data Extraction

The characteristics of the selected evidence were summarized according to what was reported in the original publications using a standardized data extraction format and were organized in chronological order. The data collected included author, year, study design, population, type of fracture, technological tool (surgical planning, intraoperative navigation, endoscopic technique, use of implants), follow-up period, assessment method and individual findings for each study.

### Data Synthesis

Due to the heterogeneity of study designs, combining techniques and the way the primary outcomes were measured; statistical pooling was considered inappropriate, and the findings were summarized narratively. We produce the 'Summary of findings' tables for characterization of the information and for the main comparisons between the results of the different combination techniques.

## Results

### Selection Process

**Fig. 1:**
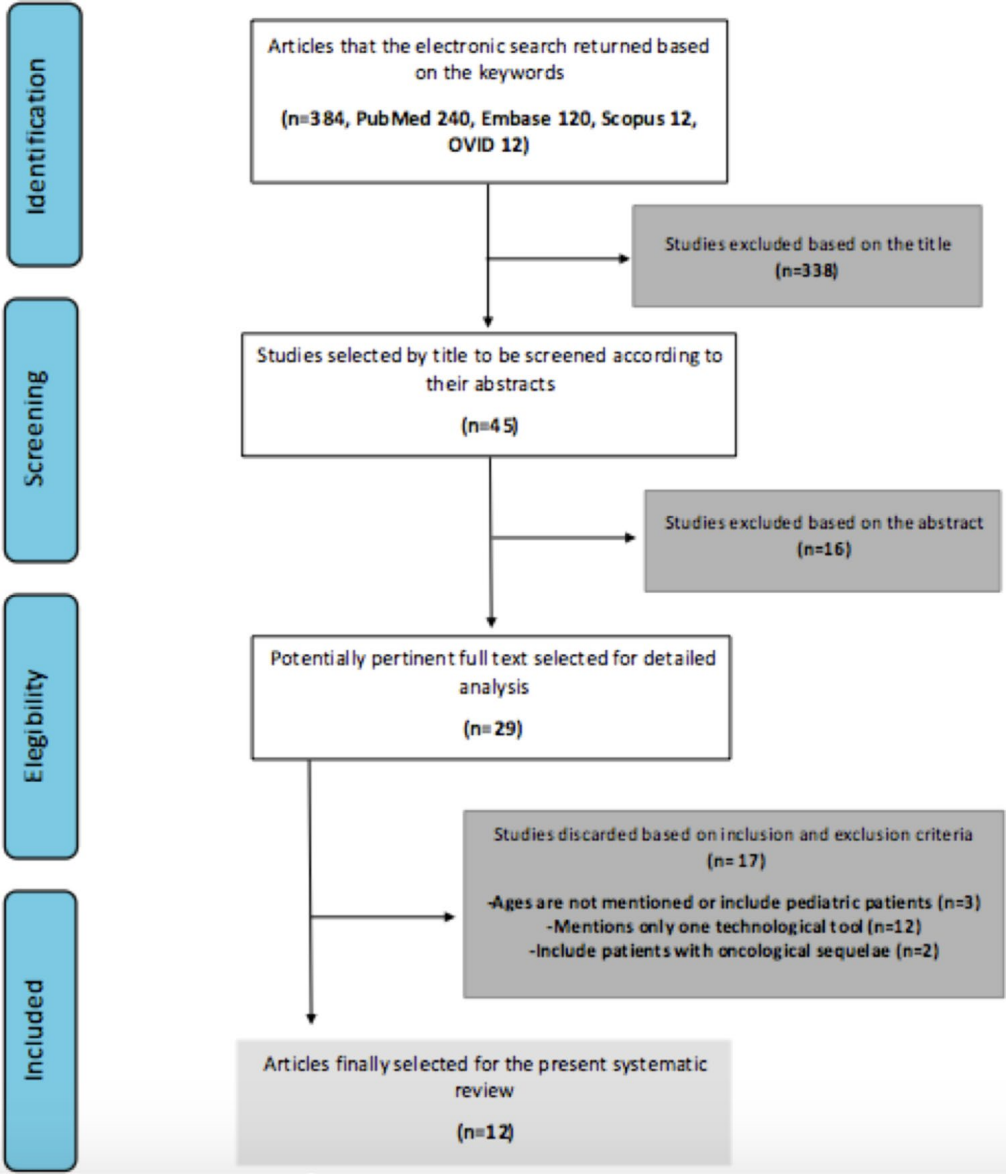
Flow diagram of the search strategy

The study selection process and summary are shown in Fig. 1. The literature review yielded 384 articles, and 338 were excluded based on the title. Then, 45 studies were evaluated for eligibility by the title and subsequently chosen to read the abstracts. A total of 16 studies were excluded based on the abstract. Twenty-nine potentially pertinent full texts were selected for detailed analysis. Seventeen studies were not considered in the end because they did not mention the age of the subjects studied, included oncological reconstructions, or did not include at least two technologies during the reconstruction to study synergism. Twelve were finally selected after reading them and applying the inclusion and exclusion criteria. A total of 418 patients were reported in the 12 studies that were reviewed.

### Description of Studies

Of the articles selected for the systematic review, 8 were case series [11–18], 2 were case reports [2, 19], 1 was quasi-experimental [20] and 1 was a randomized controlled trial [21]. All the patients underwent a reduction of the fracture that involved either the floor or the medial wall of the orbit. The follow-up was between 1 month and 13.8 months Table 1.

**Table 1:** Characteristics of included studies

References	Study design	Patients	Fracture	VP	VN	ES	PSI or RP	Follow-up (months)
Rana et al. [12]	Retrospective	100	Orbito-zygomatic	Yes	Yes	No	Yes	12
Tel et al. [15]	Case series	14	Orbital floor	Yes	No	Transmaxillary	No	6
Herford et al. [2]	Case report	1	Orbital floor	Yes	Yes	No	Yes	1.1
Yang et al. [13]	Retrospective	17	Orbital floor	Yes	Yes	Transapproach	No	24
Moura et al. [11]	Case report	1	Orbital floor	Yes	No	Transmaxillary	No	1
Chen et al. [16]	Retrospective	24	Orbital floor and medial wall	No	Yes	Transmaxillary	No	13.8
Zimmerer et al. [21]	Randomized controlled trial	195	Orbital floor and/or medial wall	Yes	Yes	No	Yes	3
Gander et al. [14]	Case series	12	Simple, complex orbital fractures and combined midface fractures	Yes	Yes	No	Yes	Not mentioned
Cha et al. [17]	Prospective	12	Orbital floor and medial wall	Yes	Yes	No	Yes	6
Podolsky et al. [19]	Case report	1	Orbital floor	Yes	No	No	Yes	1
Rana et al. [20]	Prospective	34	Orbital floor and medial wall	Yes	Yes	No	Yes	12
Copelli et al. [18]	Prospective	7	Medial wall	Yes	Yes	Transnasal	No	6

### Risk of Bias

**Fig. 2:**
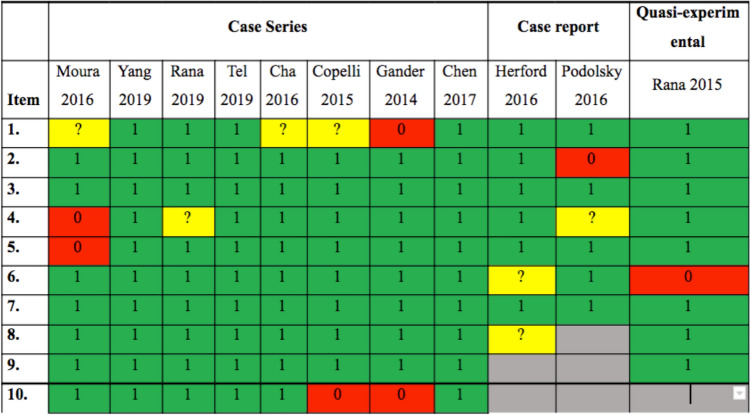
Risk of bias summary: a review of the authors' judgments using the Joanna Briggs Institute Verification List (JBI)

The articles were classified as having a high, acceptable, or low risk of bias following the Joanna Briggs Institute (JBI) Checklist. According to the analysis that was carried out, none of the articles had a high risk of bias. On the contrary, all of them were classified as having an adequate, acceptable, or low risk of bias (Fig. 2).

For Case Series, the item "Was the statistical analysis appropriate?" was not completely adequate in some studies [14, 18]. Therefore, this item in those studies was classified as having a high risk of bias. On the other hand, the domain "Are there clear criteria for inclusion in the case series?" had a high risk of bias in one study [14] and was acceptable in two studies [11, 17, 18]. However, as we already mentioned previously, all the studies analyzing all the items had a low risk of bias due to adequate methodological quality.

For case reports, one study had two items ("Was the post-intervention clinical condition clearly described?" and "Does the case report provide takeaway lessons?") with acceptable risk of bias [2]. The second study had one item with acceptable risk of Bias ("Were diagnostic tests or assessment methods and the results clearly described?") and another item with high risk of Bias ("Was the patient’s history clearly described and presented as a timeline?) [19].

In the quasi-experimental study, only one item ("Was follow-up complete, and if not, were differences between groups in terms of their follow-up adequately described and analyzed?") had a high risk of bias. Nevertheless, in general, all these previous studies had adequate methodological evaluation [20].

**Fig. 3:**
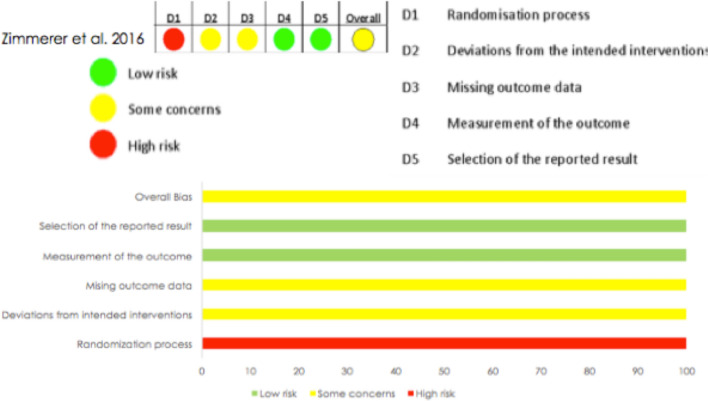
Risk of bias summary: a review of the authors' judgments using the ROB 2 tool (Version 2 of the Cochrane risk-of-bias tool)

The study by Zimmerer et al. was evaluated using the ROB 2 tool for randomization-controlled trials, obtaining poor results regarding the randomization process. This item presented a high risk of bias. Concerning domains two and three, some concerns were raised: deviations from planned interventions and missing outcome data, respectively. However, the full evaluation of the study resulted in "some concerns" about the risk of bias. [21] (Fig. 3).

### Technological Tools

The most used tool was virtual planning, which is reported in 11 (91.6%) of the articles reviewed in this work) [2, 11–15, 17–21]. The combination of technologies for the management of orbital trauma currently most used by surgeons, according to our results, was virtual planning and intraoperative navigation, which were used in 9 (75%%) of the 12 articles reviewed. [2, 12–14, 17, 18, 20, 21]

Evaluation of enophthalmos and orbital volume are the variables most frequently used in the studies analyzed in this review. However, these variables are evaluated in different ways depending on the study. In some cases, the orbital volume is compared with the contralateral orbit, and in other cases, the pre-surgical orbit is compared with the post-surgical orbit. In any case, there was a noticeable improvement in orbital volume with the use of technological tools in all the studies that included this variable. Additionally, enophthalmos were not evaluated in all studies (Table 2).

**Table 2:** Outcomes of included studies

References	Assessment method	Types of technological tools	Findings	
			Preoperative	Postoperative
Rana et al. [12]	Orbital volume and intraorbital angulation of the implant	(VP) + (VN) + (PSI or RP)	The mean (SD) orbital volume was 27.9 (4.0) cm^3^	The mean (SD) orbital volume 27.5 cm^3^ (4.1) (*t* = 0.959; p = 0.338)
Tel et al. [15]	Enophthalmos	(VP) + (ES)	Hertel scale measurements showed a mean of 0.92 mm	Hertel scale measurements showed a mean of 0.10 mm
Herford et al. [2]	Qualitative assessment- Computer Tomography	(VP) + (VN) (PSI or RP)	Not mentioned	Correct positioning of the implant and a remarkable improvement in globe position
Yang et al. [13]	Enophthalmos	(VP) + (VN) + (ES)	Average enophthalmos 2.99 to	Average measurements 0.68 mm
Moura et al. [11]	Case report	(VP) + (ES)	Not mentioned	Patient 1: Healthy orbit volume 15.309 cm^3^, Reconstructed orbit 15.318 cm^3^ (%Difference 0.06) Patient 2: Healthy orbit volume 13.677 cm^3^, Reconstructed orbit 14.503 cm^3^ (%Difference 6.04) Patient 3: Healthy orbit volume 13.309 cm^3^, Reconstructed orbit 12.740 cm^3^ (%Difference -4.26)
Chen et al. [16]	Orbital volume	(VN) + (ES)	In early reconstruction: Mean interorbital volume difference 1.72 In late reconstruction: Mean interorbital volume difference 3.41	In early reconstruction: Mean interorbital volume difference 0.53 ml In late reconstruction: Mean interorbital volume difference 0.56 ml
Zimmerer et al. [21]	Orbital volume	(VP) + (VN) + (PSI or RP)	Not mentioned	The differences in volume between the reconstructed and the unaffected orbit was 1.8 ml^2^ in patients treated with preformed and 0.6 ml^2^ with individualized implants
Gander et al. [14]	Variance implant position vs digital plan	(VP) + (VN) + (PSI or RP)	Not mentioned	Variance between 0.3 mm and 1.6 mm from implant position vs. digital plan
Cha et al. [17]	Orbital volume ratio (OVR)	(VP) + (VN) + (PSI or RP)	Mean OVR 1.0952 ± 0.0662 cm^3^	Mean OVR 0.9942 ± 0.0427 cm^3^
Podolsky et al. [19]	-Clinical evaluation -Computed tomography	(VP) + (PSI or RP)	Not mentioned	Resolution of enophthalmos, symmetrical palpebral fissure height,, excellent positioning and contour of the implant (orbital floor was completely restored)
Rana et al. [15]	Orbital volume	(VP) + (VN) + (PSI or RP)	Not mentioned	The difference in volume between the unaffected and reconstructed orbits differed significantly between the 2 study groups (PBTM: mean 0.6; SD 0.1; PSI: mean 0.4; SD 0.1; P = .029)
Copelli et al. [18]	Orbital volume	(VP) + (VN) + (ES)	Mean orbital volume 33.2 cm	Mean orbital volume 3 31 cm^3^

*VP* virtual planning, *VN* virtual navigation, *ES* endoscopic support, *PSI* or *RP* patient-specific implant or rapid prototyping, *OVR* orbital volume ratio, *CT* computed tomography, *PSI* patient-specific implant, *PBTM* pre-bent titanium mesh (NM)

## Discussion

Literature shows that complications associated with the orbital globe's position vary between 20 and 80%, possibly due to the difficulty of re-establishing the complex anatomy. But software could indicate an approximate initial anatomy [22, 23]. The enophthalmos may be related to inadequate reconstruction and the inability to re-establish orbital volume by 8.5%. Stereolithographic models and virtual planning allow for the design of patient-specific implants, reducing the risk of a poorly bent plate and reducing surgical time. On the other hand, persistent diplopia is observed in between 10 and 37% of the cases, affecting quality of life [24]. Loss of anatomical landmarks and limited space, in addition to severe trauma and extensive resections, also make eye repositioning difficult [25].

Chen et al. [26], carried out a study to observe long-term enophthalmos in orbital fractures operated with conventional methods. They mainly considered the surgical time and the walls involved as variables to study, and they observed that residual enophthalmos incidence persisted at 11.8% for single-wall fractures, 27.4% for double-wall fractures, and 16.4% for fractures involving three orbital walls [26]. Ideally, the primary management should be timely and precise and use the most considerable number of tools available to achieve the best results possible. Several studies have shown that an increase in orbital volume between 0.5 and 1 cm^3^ can cause at least 1 mm of enophthalmos after trauma [27–29].

### Integration of Technology

Although many current studies are using these technologies, only two articles have used them all. One of them, Copelli et al., in 2015, published a study that included 53 subjects with orbital fractures. The mean difference between the treated and unaffected orbits was 0.93 cm^3^. The authors suggest that endoscopic support provides a surgical approach without facial scarring and allows for adequate visualization of the surgical field. Also, intraoperative navigation increases the benefits of endoscopic support, obtaining better and more accurate results in repositioning fragments in orbital fractures [18]. Chen et al. [16] described 24 cases of orbital trauma, that resulted in diplopia and enophthalmos. They suggested the potential benefits of combining endoscopy and intraoperative navigation in primary management. This is supported by the findings that when the fractures were managed 6 months after injury, 72% of enophthalmos persisted. In contrast, when performed within the first 2 weeks, enophthalmos persisted in 20% of patients, showing a significant reduction in the resulting sequelae [16].

### Reconstruction Material

On the other hand, titanium offers advantages such as its high biocompatibility and long-term stability, but due to the anatomical complexity of the orbit, it is difficult to accurately reproduce its dimensions with this material. This is why, within its presentations, we find preformed titanium meshes and orbital implants that must be manually molded and adapted to the anatomy. In 2017, Peng et al. compared the consequences of the use of preformed titanium mesh and titanium-covered porous polyethylene sheets. The results showed no statistically significant differences in terms of complications. However, it is suggested that in cases where greater difficulty is foreseen, the use of preformed meshes is chosen due to their high capacity to reproduce volume and shape [30].

### Surgical AIDS

The PSI and VP in facial trauma, may be preferred in cases of severe destruction or tumor ablative surgery. In our search, it was only used in 6 studies for orbit trauma, and in none of the cases was it used in combination with endoscopic surgery [2, 12, 17, 19, 20].

**Fig. 4:**
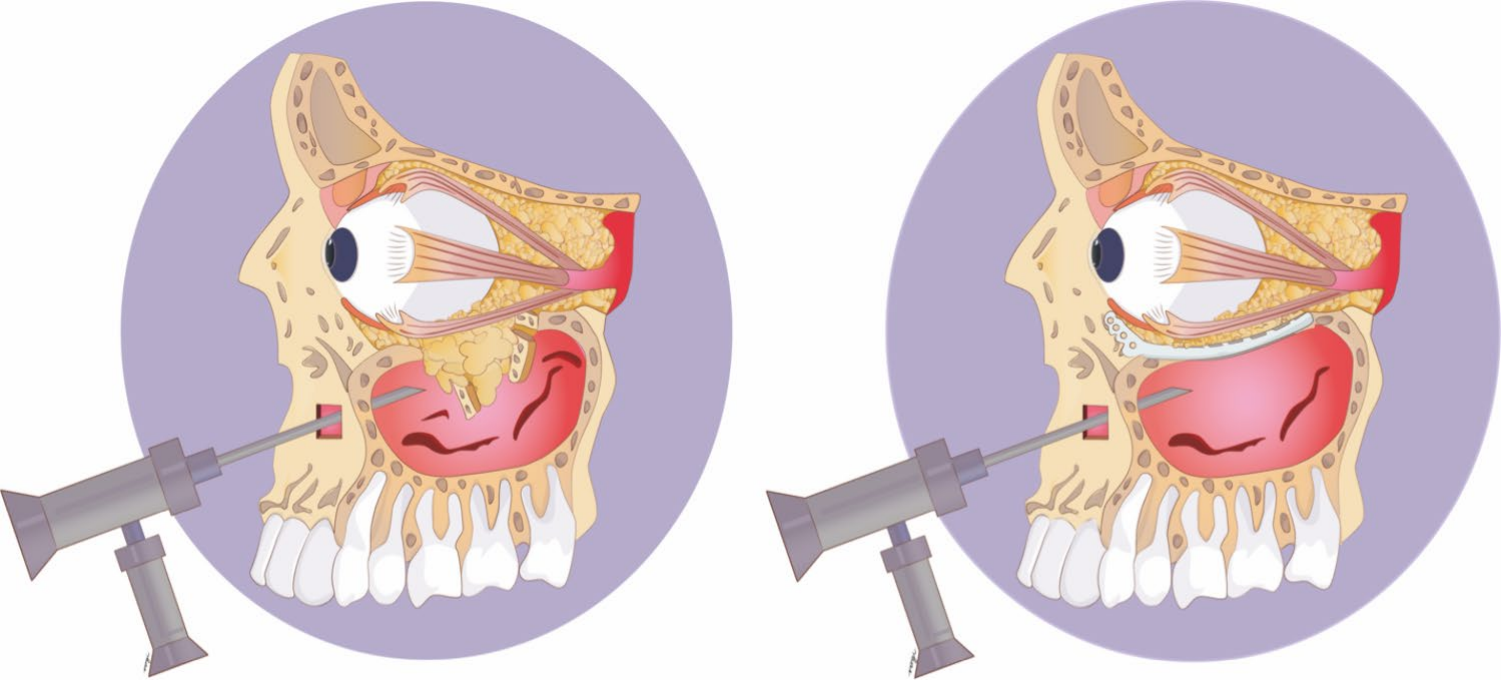
Graphic is showing endoscopic surgery with a transmaxillary approach that is very useful in the visualization of fractures and adaptation of the reconstruction meshes without the need for additional incisions in the face

Endoscopic surgery offers good results. It has already been said that good plate visibility, adequate reduction control, and a minimally invasive approach are the main virtues of the technique. A low rate of complications is also shown [31–34]. In some cases, it was used in combination with other technological tools (virtual navigation or virtual planning) [11, 13, 15, 16, 18]. For orbital floor fractures, the transmaxillary approach seems to be preferred [11, 15, 16]—Fig. 4.

Intraoperative tomography was not included in this review since it has mainly been used in the reduction of orbitozygomatic fractures by evaluating the position of the orbital floor mesh and the position of the malar. Many authors see this fundamental technology in use during the management of complex maxillofacial trauma [35]. This review focused on the technological support that helps restore the orbital volume.

The technology undoubtedly contributes to the treatment of orbital fractures; however, the study of the synergism that different techniques can have, could be a topic of discussion that requires more analysis and higher-quality studies.

## Implications for Clinical Practice

Good results were found in favor of the use of technology in orbital trauma due to more precise treatments attributed to better planning and visualization. But it is also important to consider the costs, additional training, and a wide learning curve. However, obtaining more precise reductions and having greater control of the surrounding anatomy make the use of technological tools in combination relevant in orbital trauma.

## Implications for Future Research

Even though the included studies presented adequate methodological quality, they are still mostly case series. This is why, for future research, we suggest that more randomized clinical studies be carried out to evaluate the different combinations of technological tools in orbital trauma and obtain better quality results. Additionally, due to the heterogeneity in the measurements of the variables considered in the studies, their evaluation is difficult. Therefore, we also suggest that certain evaluation measures be established to homogenize the results obtained in the different studies.

## Appendix 1: Individual search strategies

PubMed search terms: orbit* fracture OR orbit* trauma AND (computer-assisted surgery OR surgical navigation system OR navigation surgery OR endoscopic surgical procedure OR endoscopy OR patient-specific implants).

Scopus search terms: (orbit AND trauma OR orbit AND fracture) AND (computer-aided AND surgical AND simulation OR surgical AND navigation OR patient-specific AND implant OR endoscope AND support).

Embase search terms: (orbit AND trauma OR orbit) AND fracture AND (computer AND assisted AND surgery OR surgical) AND navigation OR patient) AND specific AND implant OR endoscopic) AND surgery.

Ovid search terms: (orbit fracture or orbit trauma) and (computer-assisted surgery or endoscopic surgery or patient-specific implant or surgical navigation).

## Case Series

(1) Were there clear criteria for inclusion in the case series?; (2) Was the condition measured in a standard, reliable way for all participants included in the case series?; (3) Were valid methods used for identification of the condition for all participants included in the case series?; (4) Did the case series have consecutive inclusion of participants?; (5) Did the case series have complete inclusion of participants?; (6) Was there clear reporting of the demographics of the participants in the study?; (7) Was there clear reporting of clinical information of the participants?; (8) Were the outcomes or follow-up results of cases clearly reported?; (9) Was there clear reporting of the presenting site(s)/clinic(s) demographic information?; (10) Was statistical analysis appropriate?

## Case report

(1) Were patient’s demographic characteristics clearly described?; (2) Was the patient’s history clearly described and presented as a timeline?; (3) Was the current clinical condition of the patient on presentation clearly described?; (4) Were diagnostic tests or assessment methods and the results clearly described?; (5) Was the intervention(s) or treatment procedure(s) clearly described?; (6) Was the post-intervention clinical condition clearly described?; (7) Were adverse events (harms) or unanticipated events identified and described?; (8) Does the case report provide takeaway lessons?

## Quasi-experimental studies

(1) Is it clear in the study what is the ‘cause’ and what is the ‘effect’ (i.e., there is no confusion about which variable comes first)?; (2) Were the participants included in any comparisons similar?; (3) Were the participants included in any comparisons receiving similar treatment/care, other than the exposure or intervention of interest?; (4) Was there a control group?; (5) Were there multiple measurements of the outcome both pre and post the intervention/exposure?; (6) Was follow-up complete and if not, were differences between groups in terms of their follow-up adequately described and analyzed?; (7) Were the outcomes of participants included in any comparisons measured in the same way?; (8) Were outcomes measured in a reliable way?; (9) Was appropriate statistical analysis used?
